# Investigation of ac-magnetic field stimulated nanoelectroporation of magneto-electric nano-drug-carrier inside CNS cells

**DOI:** 10.1038/srep45663

**Published:** 2017-04-04

**Authors:** Ajeet Kaushik, Roozbeh Nikkhah-Moshaie, Raju Sinha, Vinay Bhardwaj, Venkata Atluri, Rahul Dev Jayant, Adriana Yndart, Babak Kateb, Nezih Pala, Madhavan Nair

**Affiliations:** 1 Center for Personalized Nanomedicine, Institute of NeuroImmune Pharmacology, Department of Immunology, Herbert Wertheim College of Medicine, Florida International University, Miami, USA; 2 Department of Electrical and Computing Engineering, Florida International University, Miami, USA; 3 Department of Biomedical Engineering, Rutgers University, Piscataway, NJ, USA; 4 National Center for NanoBioElectronics, West Hollywood; California Neurosurgical Institute, Los Angeles; Brain Mapping Foundation, West Hollywood; Society for Brain Mapping and Therapeutics, West Hollywood, CA, USA

## Abstract

In this research, we demonstrate cell uptake of magneto-electric nanoparticles (MENPs) through nanoelectroporation (NEP) using alternating current (ac)-magnetic field stimulation. Uptake of MENPs was confirmed using focused-ion-beam assisted transmission electron microscopy (FIB-TEM) and validated by a numerical simulation model. The NEP was performed in microglial (MG) brain cells, which are highly sensitive for neuro-viral infection and were selected as target for nano-neuro-therapeutics. When the ac-magnetic field optimized (60 Oe at 1 kHz), MENPs were taken up by MG cells without affecting cell health (viability > 92%). FIB-TEM analysis of porated MG cells confirmed the non-agglomerated distribution of MENPs inside the cell and no loss of their elemental and crystalline characteristics. The presented NEP method can be adopted as a part of future nanotherapeutics and nanoneurosurgery strategies where a high uptake of a nanomedicine is required for effective and timely treatment of brain diseases.

Novel therapies for the treatment of a targeted disease are now focusing on site-specific delivery and on-demand release of a therapeutic agent^1^,^2^,^3^. Methods for the efficient intracellular delivery of a therapeutic agent using an appropriate drug nanocarrier (NC) and guided transport without causing cell damage and toxicity are both urgently required^1^,^2^. One such approach is electroporation, which opens cell membrane pores and enhance the uptake of a therapeutic agent or cargo^4^,^5^. The process is also used to navigate and guide the therapeutic material inside the cell or tissue thereby achieving maximum efficacy. The potential benefits of electroporation for targeted biomedical application have been explored for transporting nano/micro molecules^6^, DNA^7^, genes^8^, plasmids^9^, antibodies^10^, and specific drug^5^ into the cells^11^. Electroporation has shown advantages over physical methods of therapeutic delivery such as micro-injection, gene gun, laser irradiation, and sonoporation^12^,^13^. Various methodologies including high voltage electric pulse^14^, strong electric field^15^, focused laser^16^, electroplating^17^, and magnetic field^18^,^19^ are being used to perform electroporation for *in-vitro* and *in-vivo* applications^20^,^21^. In spite of successful implication with desired benefits of electroporation in therapy, the related mechanisms and its validation are not completely understood.

Recently, considerable attention has been paid toward developing pharmacologically relevant nanoformulation (NF), acknowledged as nanomedicine, to find better treatment for the targeted diseases. Nanomedicine seems to have a great future due to its two unique features, site-specific delivery and stimuli-responsive release of drug to retain maximum efficacy^22^. In combination with electroporation, NF exhibit improved efficacy and long-acting therapeutic effect due to high cell uptake through nanoelectroporation (NEP)^5^. The NEP of a therapeutic cargo is dependent on selected stimulation method, nanomaterial selected for NF preparation, and its optical, electrical, and magnetic characteristics^23^. Sometimes nanomaterials themselves show enhanced optical, electrical, and magnetic response under the exposure to stimulation, facilitating NEP phenomenon^19^. However, the understanding and demonstration of this phenomenon need to be studied and explored in more detail. There is a considerable scope to explore 1) smart drug-NCs able to stimulate NEP, 2) new methodologies for NEP without affecting biological activity of therapeutic agents and cell viability, and 3) advanced techniques to evaluate cell uptake, in particular, intracellular distribution and chemical integrity of NCs.

Intracellular delivery of metal-NCs for various biomedical applications has been demonstrated. However, this is relatively different from polymer NCs because the leaching of metal NCs, and heating produced by metal NCs on external stimulation by light, magnetic or electric field, are two major concerns when developing metal-based nanomedicine^24^,^25^. Bhardwaj *et al*.^24^ demonstrated a conventional electroporation using BioRad’s micropulser to deliver metal NCs inside the cell^24^. Surprisingly, in presence of metal NCs a severe cellular damage and toxicity was observed even at lowest achievable electroporation parameters, single pulse of 1 ms with electric field strength of 0.5 kV cm^−1^ 24. Thus, optimization of electroporation to achieve efficient intracellular delivery of a therapeutic cargo is challenging but critical and immediately required^26^,^27^.

Among the various metal drug-NCs, magneto-electric nanoparticles (MENPs) composed of BaTiO_3_@CoFe_2_O_4_, have been recently explored for drug delivery to treat targeted diseases^1^. The MENPs are monodispersed cationic magnetic core-shell particles with the average size of 25 nm and do not cause cellular toxicity^1^. Specially designed ferromagnetic core-shell structure of MENPs exhibited polarization on applying external ac-magnetic field, which is capable to breaking of electrostatic bonds between MENPs and drugs^5^,^22^. The potentials of MENPs for on-demand drug release to cure targeted diseases with future prospects have been described by Kaushik *et al*.^28^. Nair *et al*. have demonstrated delivery and on-demand release of anti-HIV drug using MENPs across the blood-brain barrier (BBB) to cure neuroHIV^22^. Similar approach has also been used for the delivery and release of Beclin1-siRNA^29^. The magnetically guided brain delivery of MENP in mice model has successfully been demonstrated recently by Kaushik *et al*^1^. The results of this study confirmed uniform distribution of MENPs in the brain, safe for major organs (brain, liver, lung, spleen, and kidney), non-toxic blood profile (renal and liver function), and procedure did not affect motor coordination function^1^. Another highly significant aspect of MENPs, as drug-NC for the treatment of cancer, was explored by Guduru *et al*.^5^ using a NF of MENP carrying Paclitaxel. The tumor, based on human ovarian carcinoma model, was eradicated within 24 hrs. In this approach, externally applied dc-magnetic field to MENPs facilitated electroporation in tumor cell membrane and resulted in a significant cell uptake of NF^5^. This methodology showed significant reduction in tumor size when used on mice xenografts^23^. Authors proposed NEP was responsible for high cell-uptake of developed NF, although related mechanism and validation of NEP was not demonstrated.

To answer this fundamental question and to investigate a more effective cure for brain diseases, we explored NEP of MENPs by applying ac-magnetic field inside microglia (MG) cells, *in-vitro*. This type of brain cell is sensitive to CNS infectious diseases, including for example HIV/AIDS. The presence of MENPs inside the brain cell was confirmed using focused-ion-beam assisted transmission electron microscopy (FIB-TEM) analysis. The FIB is the most advanced technique adopted recently for the sampling of biological materials using cryo-electron microscopy^30^,^31^,^32^,^33^,^34^,^35^,^36^. It is especially useful with transmission electron microscopy (TEM)^30^ because the biological structure of cells and tissues can be explored during therapy and treatment progression. The method is completely automated and has demonstrated advantages of time and labor over ultramicrotomy^30^,^33^. Therefore, FIB was used for slicing MG, after NEP of MENPs, for TEM experiments. The experimental results were validated using a numerical simulation model.

## Results

### Characterization of MENP

**Figure 1:**
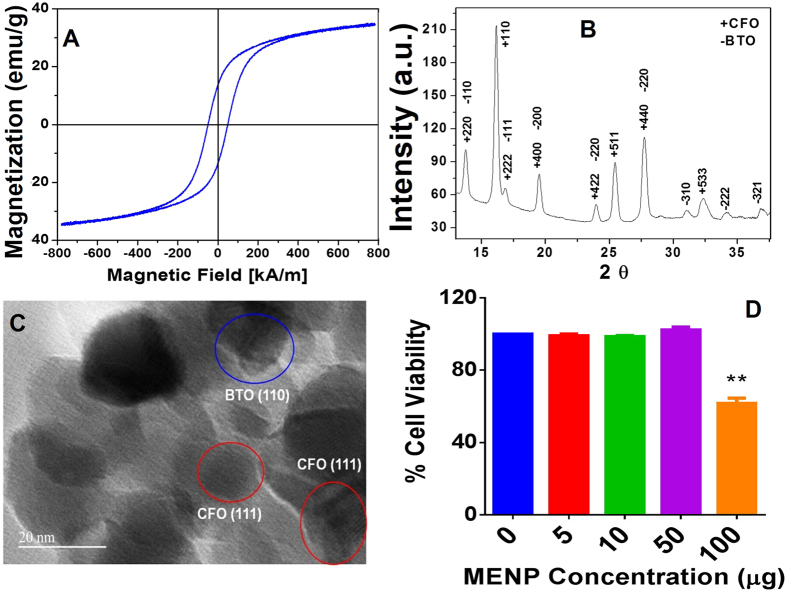
Characterization of MENPs. (**A**) VSM hysteresis confirm ferromagnetic nature of MENPs, (**B**) XRD pattern confirm that MENP are crystalline and composed of CFO and BTO, (**C**) TEM images confirmed particles size of MENPs as 25 ± 5 nm, and (**D**) MENP dose dependent cytotoxicity evaluation using MG brain cells, results confirmed that 10 μg MENP are safe to perform planned experiments.

The cationic surface charged ferromagnetic (Fig. 1A) MENPs are crystalline and composed of BaTiO_3_ (BTO) and CoFe_2_O_4_ (CFO) phases (Fig. 1B) with estimated average size of 25 ± 5 nm (Fig. 1C). The MTT [3-(4,5-Dimethylthiazol-2-yl)-2,5-Diphenyltetrazolium Bromide]-assay based cytotoxicity assessment with respect to MG cells types (1 × 10^6^ cells) confirmed that MENPs ≤50 μg are biocompatible (cell viability > 90%) (Fig. 1D). The higher concentration of MENPs ≥50 to 100 μg showed less cell viability (~65%). Cytotoxicity assessment findings confirmed that MENPs dose <50 μg are safe for experiments. To avoid aggregation and considering minimum detectable dose, we selected 10 μg of MENPs for NEP experiments.

### Demonstration of MENP-NEP in MG cells, *in-vitro*

**Figure 2:**
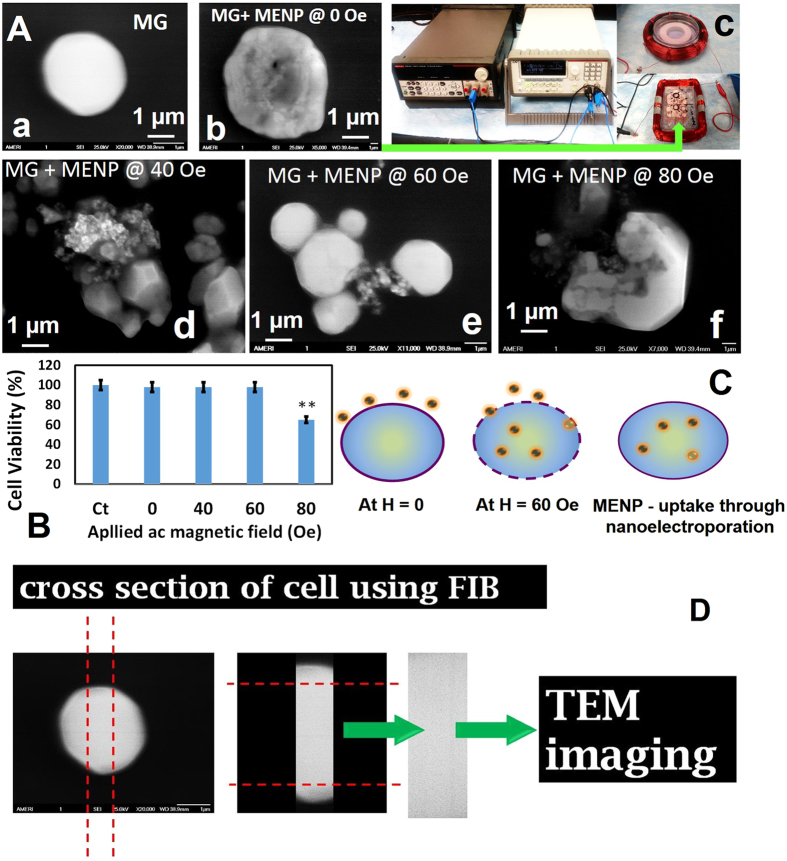
Electroporation of MENP, *in-vitro* MG model. (**A**) SEM image of MG grown onto Au chip (a), morphology of MENP absorbed onto MG surface (b), demonstration of electroporation stimulated by ac-magnetic field applied through electromagnetic coil (c), SEM image of MG + MENP, after electroporation at applied magnetic field of 40 Oe for 30 minutes (d), SEM image of MG + MENP, after electroporation at applied magnetic field of 60 Oe for 30 minutes (d), SEM image of MG + MENP, after electroporation at applied magnetic field of 80 Oe for 30 minutes (d). (**B**) MTT-assay based cytotoxicity evaluation of MG + MENP with and without applied ac-magnetic field of various strength. The cell viability % was at every experiment condition was compared with MG as control. (**C**) Proposed mechanism of electroporation stimulation by ac-magnetic field caused MENP surface potential changes and distortion of phospholipids layer of MG cells resulting in cell-uptake of MENP inside the cell. (**D**) Strategy to confirm electroporation through FIB assisted TEM. The MG cells grown on to Au chip will be used for FIB milling and further used for TEM experiment to confirm the MENP distribution and chemical integrity within cells.

For NEP, ac-magnetic magnetic field of varying strength (40, 60, and 80 Oe) was applied on MENP (10 μg)/MG/Au-chip through electromagnetic coil. Morphological assessment of MG cells in presence of MENP with and without ac-magnetic field stimulation was performed using SEM (Fig. 2A(a,b,d–f). The MG cells uniformly grown onto Au chip exhibited very smooth surface morphology (Fig. 2A(a)). In presence of MENPs, an adsorption of MENPs were observed on the surface of MG cells due to electrostatic interactions (Fig. 2A(b)). Upon applying ac-magnetic field by an electromagnetic coil (Fig. 2A(c)), at 40 Oe (Fig. 2A(d)), an increased accumulation of MENPs were observed due to enhanced polarization in MENPs. In addition, ac-magnetic field generated charge difference on the cell surface. At 60 Oe, well-arranged MENPs on MG cells surface were observed (Fig. 2A(e)). This may possibly be due to gradual navigation of MENPs inside the cells. This explanation is in agreement with a numerical model reported by Betal *et al*.^19^. The results of their numerical simulation study confirmed that the ferroelectric core of MENPs (CoFe_2_O_4_) exhibits a deformation on ac-magnetic field stimulation due to its magnetostriction characteristics. This shape deformation generates a magnetoelastic pressure wave which is absorbed by the piezoelectric shell i.e., BaTiO_3_ of MENP. The piezoelectric shell converts pressure waves to a change in surface potential. The repeated surface potential change on MENPs generates pulsed negative localized surface potential. This process takes place repeatedly under ac-magnetic field and at sub-micrometer distance from the cell boundary, which leads to phospholipid position dislocation^19^. Eventually, electrical pulses from MENPs results in dislocation of phospholipids in the hydrophilic region to form nanopores in the cell membrane i.e., NEP. Overall, on ac-magnetic field exposure, MENPs will continue to penetrate the membrane through the electrically induced nanopores due to special core-shell structure. Results of reported numerical model and our experimental findings confirm MENP as NEP facilitator on ac-magnetic field stimulation. At higher ac-magnetic field, 80 Oe, considering the similar mechanism, the presence of MENPs inside MG cells can clearly be seen (Fig. 2A(f)). However, at this magnetic field the morphology of MG cells found to be deteriorated significantly due to the toxicity caused by heat generated at high ac-magnetic field.

It’s well known that most of the stimuli-responsive electroporation forces may cause cell damage through toxicity^37^. The MTT-assay (Fig. 2B) results confirmed that MENPs and ac-magnetic field at 40 Oe and 60 Oe did not affect the cell viability (96 to 98%) in comparison of control MG cells (100%). However, the cell viability of MG cells reduces to 65% at 80 Oe ac-magnetic field, applied for 30 minutes. This may be due to denaturation of MG cell wall caused probably by heat generation due to localized electric field on applying ac-magnetic field of 80 Oe or higher. These findings confirmed, that optimized ac-magnetic field to achieve safe NEP is 60 Oe, as illustrated in Fig. 2C and is validated using a numerical simulation model. To confirm the presence of MENP inside the MG cells, we studied the morphology of the cross section of MENPs-MG using FIB-assisted TEM, as illustrated in Fig. 2D.

### MENP-MG sectioning using FIB and TEM for NEP confirmation

**Figure 3:**
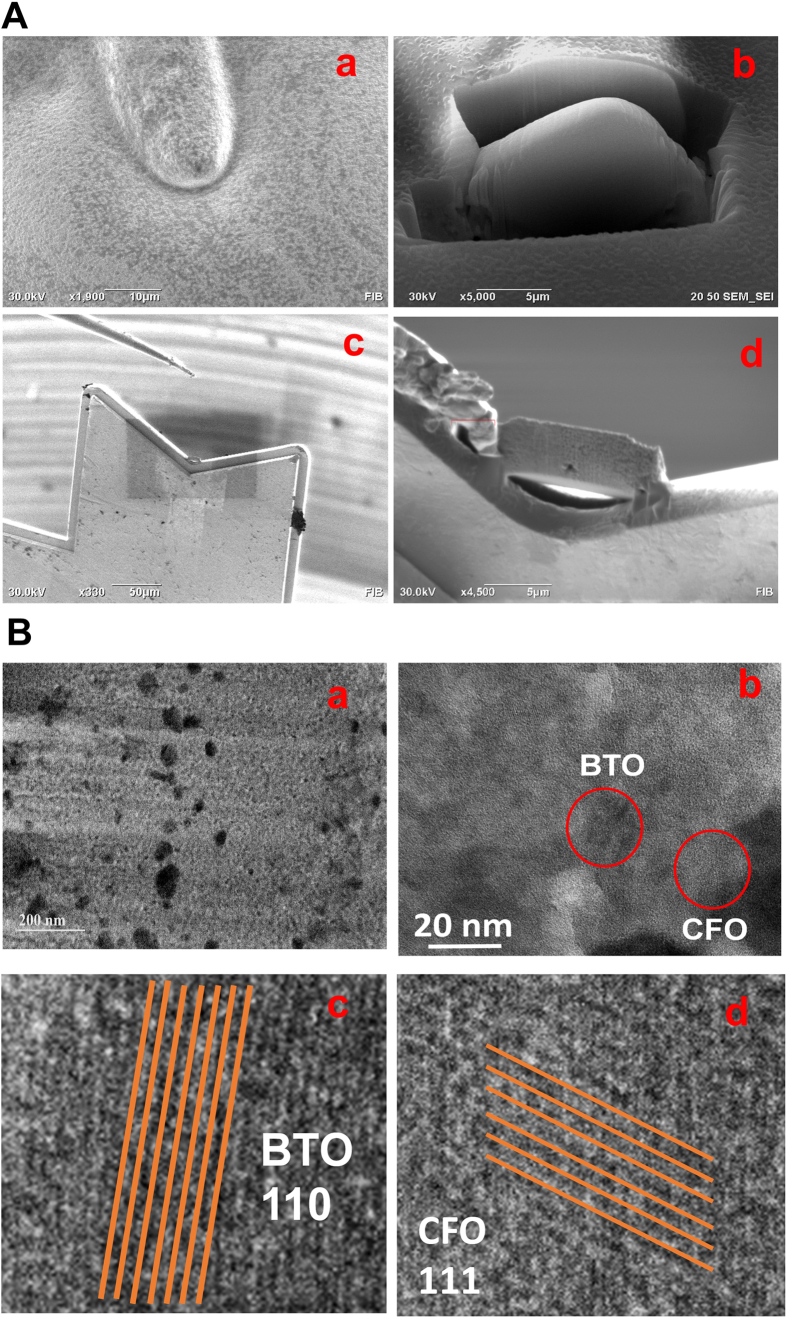
(**A**) Preparation of a thin section of a MG cell inside FIB. (a) MG cell, (b) preparation of a thin section by lift out method, (c) nanomanipulator to lift up the lamella and then weld the lamella on TEM grid inside FIB, (d) lamella of MG cell is welded on TEM grid. (**B**) TEM images of the prepared lamella of MG cell. (a) TEM image confirms that MENPs ate distributed inside the cell without agglomeration and average particle size of 25–30 nm, (b) atomic planes, as red circles, correspond to CFO and BFO, (c) BTO 110 planes, and (d) CFO 111 planes.

A FIB microscope was used to slice a thin section, around 100 nm, of MENPs nanoelectropated MG cell (Fig. 3A). The gallium ion was used to cut through the cell and create two trenches (Fig. 3A(b)). The lamella was also cut out from the bottom by gallium ion. Then, a nanomanipulator was used to lift out the lamella and to weld it to a TEM grid inside FIB (Fig. 3A(c and d)). The lamella, thin section, was used for TEM study to confirm MENP presence inside the MG cells. The TEM image (Fig. 3B(a)) showed a clear distribution of MNEPs inside the MG cell without considerable aggregation. The particles size of MENPs inside MG cells was estimated as 25 ± 5 nm, confirming that ac-magnetic field did not affect the shape and particles size of MENPs compared to the original MENPs. The NEP is a stimulus process wherein the applied force and cellular environment may alter the chemical composition and physical characteristics of a nanomaterial. To evaluate the effect of ac-magnetic field on MENPs, we performed further TEM experiments on the lamella of MG to explore regions of interest. Fig. 3B(b) exhibits 110 and 111 atomic planes correspond to BTO (JCPDS 04-001-7269) and CFO (JCPDS 00-022-1086), indicated in circles and magnified in images Fig. 3B(c and d), respectively. The results of TEM studies confirm that MENPs maintain crystalline structure and integrity throughout the process of NEP. Overall, the results of TEM studies confirms that MENPs, without disintegration, penetrate MG cells wall through NEP under ac-magnetic field stimulation which is validated using a numerical model described in next section.

### Numerical simulation modelling for NEP validation

**Figure 4:**
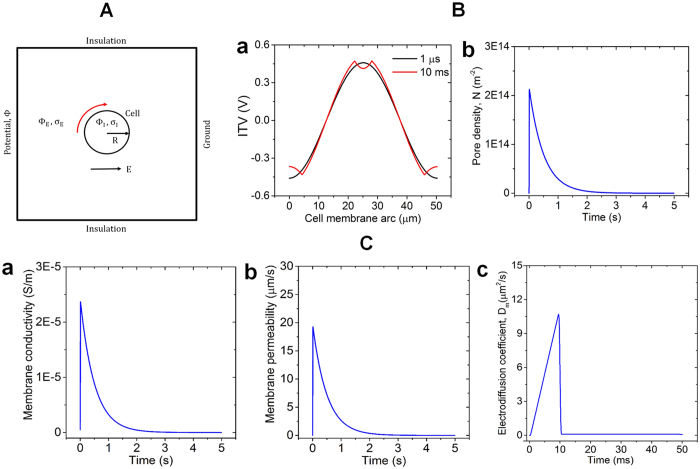
(**A**) 2D model developed in COMSOL. Schematic of the model created in COMSOL defining the boundary conditions of the external medium. Electric field is considered from left to right aligned with the x-axis direction. (**B**) Creation of membrane pores with induced transmembrane voltage. (a) The ITV profile along the membrane at two different times of the simulation (b) Cell membrane pore density over the simulation time. (**C**) Conductivity, permeability and electro-diffusion characteristics observed under 10 ms electric pulse of an external field. (a) Conductivity of the cell membrane, which reach to a maximum value of 2.36 × 10^−5^ S/m, before the pulse ceases (b) Permeability of the cell membrane also follow the same exponential trend to decrease over time after the pulse was turned off (c) Time course of electro-diffusion coefficient of the cell membrane.

A finite element method (FEM) based simulation tool, COMSOL Multiphysics 4.3 b, was used for the numerically study of NEP phenomenon of MENPs across the cell membrane through ac-magnetic field based electrically induced nanopores (Fig. 4). For better computational efficiency, a 2-D model was developed considering a spherical cell with radius of 8 μm and membrane thickness of 5 nm placed in an extracellular medium, represented by a rectangle with dimensions of 160 μm × 160 μm. The boundary conditions assigned to the rectangle are shown in Fig. 4A. The left and right side of the rectangle are taken as the positive anode and the negative cathode electrode, respectively. Considering the required mesh density for each domain in COMSOL, inclusion of an extra domain with 5 nm small region for cell membrane thickness is not only impractical but also computationally expensive. Hence, cell membrane was modelled as boundary condition in the simulation^38^,^39^. The “contact impedance” boundary condition, available in COMSOL under the electric currents of AC/DC module, is meant to approximate a thin layer of material that impedes the flow of current normal to the boundary, but does not introduce any additional conduction path tangential to the boundary. Hence, in this model, we chose and assigned “contact impedance” mode boundary condition to define the cell membrane. The numerical values of the parameters used in this simulation model along with the general parameters and rate constants are presented in Table 1.

In the experiment, with the presence of external magnetic field, MENPs generate localized electric fields near the cell membrane, which are large enough to create membrane pores allowing MENPs entering the cell. Moreover, both the electric charge density and effective magnetic charge density are amplified at the edges of MENPs due to the cubic symmetry of the real life nanoparticles, which cause enhancement of the localized field, known as edge effect. By taking into account this edge effect of MENPs, approximately 1 kV/cm magnitude of electric field can be generated in the vicinity of MENPs by applying 100 Oe external magnetic field 5. In the experiment, we found 60 Oe to be the optimum magnetic field to create successful NEP without causing any damage to the cell. Hence, in the simulation we applied an external electric field of 600 V/cm with a pulse duration of 10 ms to the positive anode electrode. We used COMSOL’s *rect* function to create the electric pulse. Electric potential of intracellular medium, Φ_I_, and extracellular medium, Φ_E_, was calculated by solving the following equation in Time Dependent Study application mode under Electric Currents of the AC/DC module in COMSOL.


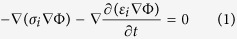


where, *σ*_*i*_ and *ε*_*i*_ are the electric conductivity and relative permittivity of the material in each domain. Then, intracellular and extracellular media was coupled on the cell membrane boundary by feeding the solutions achieved from Eq. (1) into the following equation,


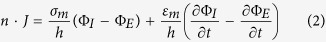


where, *n* is the unit vector normal to the cell membrane boundary surface, *J* is the current density, *σ*_*m*_, *ε*_*m*_ and *h*^40^,^41^,^42^,^43^ are the electric conductivity, dielectric permittivity and thickness of the membrane, respectively. Finally, induced transmembrane voltage (ITV) was calculated as the electric potential difference between each side of the cell membrane. The ITV obtained from the simulation along the cell membrane arc starting from the left anode pole is presented in Fig. 4B(a) at two different time of the simulation, *t* = 1 μs and *t* = 10 ms, respectively. The ITV at 1 μs shows a co-sinusoidal shape, pertinent with a cell membrane, which is not yet permeabilized. As the time increases, conductivity of the membrane around the anode and cathode poles start to increase, which results in a depression in the ITV profile around the polar regions, as shown in the Fig. 4B(a), when time is at 10 ms. This non-uniform behavior in ITV ensures that the cell is permeabilized.

In the experiment, when MENPs are within few nanometers from cell, external electric field of the cell is changed in the vicinity of the cell membrane due to the electric field pulse generated by MENP under the influence of external ac-magnetic field, which primarily results in a change in the ITV of the cell. The change in ITV causes the repulsion and dislocation of the phospholipids of the cell membrane, which finally contributes to the development of pore formation in the membrane and makes it partially permeable. This pore formation can be characterized by using a pore dynamics model, which is governed by the following relation^41^,


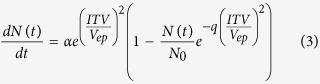


where, *N* is the induced pore density in the cell membrane, *N*_0_ is the pore density in the non-electroporated cell membrane and *q, α*, and *V*_*ep*_ are the electroporation process characteristics parameters. In COMSOL, Eq. (3) was incorporated through *Weak Form Boundary PDE* application mode under the *PDE* module. This module solves the equation and calculate the pore density created in the membrane at each time step of the simulation. Cell membrane pore density with time is presented in Fig. 4B(b). It is clearly observed that membrane pore density gets increased until the end of the electric pulse and reaches a maximum value of 2.12 × 10^14^ m^−2^. After the cessation of the pulse, membrane pores start to close up, which does not happen instantly, instead it follows an exponential path to decrease over time.

With the formation of pores in the cell membrane, conductivity of the membrane increases, which is clearly reflected in the following equation^41^,


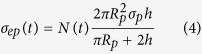


where, *N(t*) is the pore density obtained from Eq. (3), *R*_*p*_ and *σ*_*p*_ are the radius and conductivity of a single nano pore, respectively. Hence, in the AC/DC module, we expressed the total membrane conductivity as a variable *σ*_*m*_, which is the sum of passive membrane conductivity without electroporation *σ*_*m0*_ and induced membrane conductivity due to electroporation, *σ*_*ep*_. Conductivity value of the membrane was updated at each time step with the pore density achieved from Eq. (3) and then, the calculated conductivity was fed into Eq. (2), which in turn changed the value of ITV. As soon as the ITV changes, it affects the number of pore density created in the membrane, which again affects the cell membrane conductivity. This whole process went on as long as the electric field pulse was on. Conductivity of the cell membrane obtained from the simulation over the course of time is presented in Fig. 4C(a). It is observed that the membrane conductivity reaches to a maximum peak value of 2.36 × 10^−5^ S/m, whereas, the passive cell membrane conductivity was 5 × 10^−7^ S/m before the NEP of the cell.

As the cell gets permeabilized, MENPs start to pass through the pores by mainly, two mechanisms, which are diffusion and electrophoresis. Diffusion coefficient of the cell membrane contributed by diffusion mechanism only, is related to cell membrane permeability, *P*_*m*_ and can be written as,


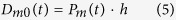


Using the pore-density obtained from Eq. (3), permeability of the membrane can be calculated by the following formula,


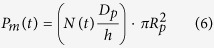


where, D_p_ is the diffusion coefficient of MENPs. Permeability behavior of the membrane is presented in Fig. 4C(b) and it is seen that it follows the same exponential path to decrease after the cessation of electric pulse. The second mechanism, electrophoresis is known as the movement of charged particles under the presence of external electric field. Diffusion coefficient contributed by both the diffusion and electrophoresis can be called electro-diffusion coefficient, which is determined by the following equation,


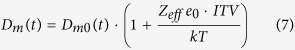


where, *Z*_*eff*_ is the effective charge number of MENPs, *e*_*0*_ is the elementary charge, *k* is Boltzmann constant and *T* is the temperature. These transport mechanisms of MENPs governed by Eqs (5, 6, 7) were implemented in COMSOL using *Chemical Species Transport* module, where cell membrane was defined by *Thin Diffusion Barrier* boundary with diffusion coefficient, *D*_*m*_(*t*), which takes into account both the diffusion and electrophoresis. This cell membrane diffusion coefficient was updated at each time step through the whole simulation with pore density value obtained from Eq. (3). The initial concentration of MENPs in the extracellular and intracellular media were set to 1 mM and 0, respectively. The simulated electro-diffusion coefficient of the membrane under the presence of field pulse is shown in Fig. 4C(c). This increases linearly over time until the pulse is turned off. By the end of the pulse it drops almost instantly to a very smaller value and from there, it decays exponentially until all the membrane pores are closed.

**Figure 5:**
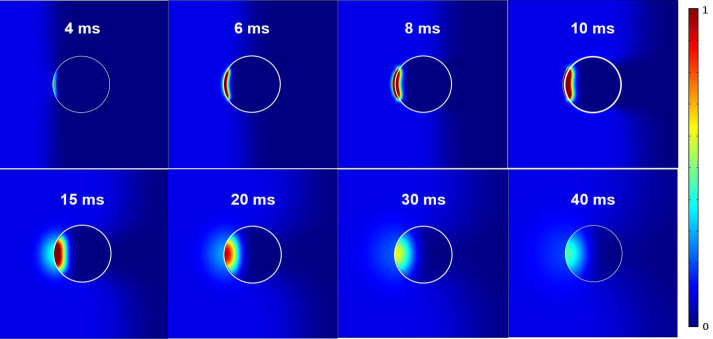
MENPs transport inside the cell. Evolution of MENPs concentration in course of time.

Finally, in Fig. 5, we present the simulation results showing the evolution of MENPs transport inside the cell in course of time due to NEP. It is clearly seen that MENPs penetrate the cell from the anode-facing side through the electrically induced nanopores during the first 10 ms. The spreading of MENPs concentration profile is primarily governed by electrophoresis for t < 10 ms, and by diffusion afterwards. During the first 10 ms, the peak concentration of MENPs inside the cell keeps increasing, as observed by the increase in intensity until 10 ms in Fig. 5. This is mainly because of the presence of electric field pulse, which creates the nanopores to open up in the cell membrane and causes the MENPs penetrating the cell due to the increase in conductivity, permeability and eventually electro-diffusion coefficient of the cell membrane as seen in Fig. 4C. After the cessation of the pulse, MENPs slowly diffuse away from the anode pole inside the cell, as seen by the decrease in intensity from 15 to 40 ms in Fig. 5. This is due to the fact that, at the end of the electric field pulse all the generated nanopores in the cell membrane are sealed up, which causes no more cellular uptake of MENPs, and the driving force of diffusion acts on all the penetrated MENPs inside the cell and eventually they reach to an equilibrium concentration. Thus, obtained numerical simulation results explain and validate that MENPs penetrate cell membrane of brain cells through electrically induced nanopores on ac-magnetic field stimulation.

## Discussion

Eelectroporation is a simple, effective, and widely accepted approach to increase cellular uptake of the therapeutic cargo. Bhardwaj *et al*.^24^ reported the heating effect from metal nanoparticles to be a critical concern and require an extensive optimization of conventional electroporation in cuvette-based design to develop a non-toxic method. Additionally, the conventional cuvette-based electroporation apparatus as well as emerging microfluidics electrode designs are only limited to *in-vitro* demonstrations^44^. The cell-uptake of carbon nanotubes inside MG (tumor macrophages) on intra-tumor injection in mice was demonstrated for real time imaging to monitor surgery^45^,^46^ acknowledged as nanoneurosurgery^47^. *In-vivo* electroporation using electrodes or plates, and electrical-stimulation has been developed including delivery of the genes to brain space^48^. However, like others, every *in-vivo* electrode-based design requires extra tools and have inherent limitation of poor field distribution and space restriction as the electroporation effect and the consequent cargo delivery will decrease from the surface towards the center^49^. Consequently, peripheral tissue, for example, sensor neurons in the outer surface of the brain will experience higher stress/toxicity as compared to inner core. On the other hand, MENPs-NEP approach presented here does not require additional apparatus to achieve electroporation *in-vivo*. In addition, the NEP will not be spatially restricted because this process is facilitated by MENPs “can be acknowledged as nanoelectroporators” uniformly distributed in the brain^1^.

The NEP, a stimulus-dependent mechanism to open cell membrane pore, has been adopted to deliver therapeutic agents inside the cells for better treatment. Site-specific drugs, DNA, gene, plasmids etc. have been administrated within cells or tissues through electroporation for better efficacy of therapeutic agents. Various methodologies are in practice and continuous efforts are being made to make them safe and adoptable at large scale needed for personalized treatment. Recently, navigation of therapeutic agents in high concentration inside cell has become possible through using a suitable nanomaterial as a carrier. These nanomaterials are particularly designed, surface charged and are biocompatible allowing them to bind strongly with the drug and offer stimuli-responsive release^4^,^28^. Nanomedicine, an optimized NF of better efficacy, is specially designed within restricted size domain (most desirable ~100 to 200 nm) so that cell penetration through NEP can be achieved easily in high concentration^5^. Such of few NFs designed using MENPs as a suitable drug- NC have been described in literature^1^,^5^,^22^,^28^.

The MENPs, a suitable drug-NC for the brain delivery^28^ bind with drug molecules e.g., anti-HIV drug^22^, anti-cancer^5^, and SiRNA^29^ via electrostatic interaction due to charge difference between particles and drugs. Due to magnetic characteristics of NF containing MENPs can across the BBB to cure neuroHIV/AIDS under the static magnetic field exposure. Recently, MENPs magnetically guided to the mice brain^1^ without compromising structural integrity and chemical composition^50^. A uniform distribution of MENPs without aggregation was observed in the brain without causing organ and blood related toxicity^1^. This non-invasive brain delivery method also did not affect the sensorimotor coordination function of mice^1^. The on-demand release of therapeutic agents form MENP based NF was demonstrated on applying ac-magnetic field (40 to 60 Oe and 1 KHz, exposure time 30 minutes)^5^,^22^,^29^. The ac-magnetic field causes polarization in MENPs resulting in surface charge distortion and finally releases the drug after breaking of electrostatic bonds. Guduru *et al*. developed a model to eradicate tumor using a MENP based NFs. Authors delivered MENP/anti-cancer-drug inside cell through electroporation on applying dc-magnetic field and further applied ac-magnetic field stimulation to release the drug^5^. This research group demonstrated this technique in mice, a significant reduction in tumor size was observed due to cell uptake of NF and on-demand release of anti-cancer drug^23^. These research findings are remarkable, but the need of two kinds of magnetic fields of different nature, dc-magnetic field for electroporation and ac-magnetic field for on-demand release, make it complicated. Thus, we believe that developing one stimulation method capable of performing electroporation and on-demand release will be of high importance. Recently, Beclin1-siRNA was delivered across the BBB using MENPs as a NC and on-demand release using ac-magnetic field stimulation (60 Oe at frequency of 1 KHz)^29^. The released Beclin1-siRNA exhibited a better efficacy in terms of regulating autophagy and inflammation during HIV-infection. This better efficacy may be due to high cell uptake of MENP/Beclin1-siRNA therapeutic cargo through electroporation, facilitated by ac-magnetic field. However, the mechanism related with this justification was not demonstrated. In agreement to our hypothesis, Betal *et al*., recently demonstrated magnetically controlled electroporation of MENP by the mechanism of magneto-elasto phenomena using human epithelial cells (HEP2)^19^. They simulated magnetic field dependent cell uptake characteristics of MENP based on magneto-elasto-electroporation mechanism. In a suspension of MENP and HEP2 cells, applied ac-magnetic field rapidly changes the surface potential of MENP and also changes transmembrane voltage of HEP2 cells resulting in opening of cell membrane i.e., nanopores due to displacements of phospholipids. The penetration of MENP across the cell membrane was confirmed using a FITC based qualitative microscopic assessment. However, numerical simulations of MENPs delivery inside the HEP2 cells under the influence of applied magnetic field was not performed^19^. In addition, the effect of ac-magnetic field on bio-distribution, toxicity, and MENPs structure were not investigated in the aforementioned study.

In conclusion, we demonstrated NEP of MENPs inside the brain cell on ac-magnetic field (60 Oe at 1 kHz). The NEP found dependent on applied ac-magnetic field in magneto-electric property of nanoparticles. The results of FIB-TEM analysis confirmed MENPs distribution inside the brain cells with original structural integrity. Experimental observations confirming NEP of MENP were in agreement with a numerical simulation model. The results of presented research confirmed that the ac-magnetic field induced NEP of MENPs is safe and can potentially be used for clinical applications.

## Methods

### *In-vitro* cell model

An *in-vitro* model was developed to perform NEP on ac-magnetic field stimulation. In this model, gold (Au) chip (2 × 2 cm), was placed at the bottom of the culture well plate and let MG (1 × 10^6^ cells) allow to grow (37 °C for 48 hrs) on top of chip surface. The MG-Au, *in-vitro*, platform is of choice to perform to perform NEP, SEM, and FIB-assisted TEM studies.

### NEP of MENPs, cytotoxicity and SEM experiments

The MENPs used in this research work was synthesized using our previously well-established multi-step synthesis method and characterized using VSM (for magnetization), XRD (crystalline structure and purity), Raman (functionality), and TEM (particles estimation as 25 ± 5 nm)^1^. The optimized amount of MENPs (10 μg) were dispersed in a well containing 1 mL culture media and pre-placed MG grown Au chip (2 × 2 cm) at the bottom. The whole set-up was placed in an indigenously designed electromagnetic coil (dimension 4 × 6 inches) connected with a wave function generator and a power supply. For NEP through stimulation via ac-magnetic field, various field of 40, 60, and 80 Oe at a constant frequency of 1 KHz was applied for 30 minutes of exposure. Our previous work demonstrated that frequency does not affect the localized electric field of MENPs and cells viability^22^. However, applied ac-magnetic field (60 Oe) at 1 kHz exhibited change surface charges of MENP required for on-demand release of drug^29^. Therefor we selected constant frequency of 1 kHz and varying ac-magnetic field for NEP. Each experiment was performed three times and proceed for further experiment. As claimed, NEP is a function of applied magnetic field and exposure time. Our groups previously has demonstrated on-demand release of Anti-HIV and anti-cancer drug on ac-magnetic field stimulation for 30 minutes. Thus we used 30 minutes as optimized time for NEP at different ac-magnetic field.

Biocompatibility is a major concern while selecting a nanomaterials for biological application. We performed MTT [3-(4,5-Dimethylthiazol-2-yl)-2,5-Diphenyltetrazolium Bromide]-assay using a standard procedure as described in our previous publication^1^. To estimate cell viability percentage at various dose of MENPs with respect to MG cells types and at every applied conditions selected for each electroporation experiments.

The MG grown onto Au chip with or without NEP were examined through SEM [JEOL 6330 F Field Emission Scanning Electron Microscopy (FEG-SEM)] to evaluate morphological changes throughout the process.

#### FIB and TEM experiments

A JEOL JIB-4500 dual beam FIB was used to prepare the thin section out of MENPs nanoelectroporated MG cells grown on Au substrate by lift-out method. A FEI CM-200 transmission electron microscope was employed to conduct TEM experiments.

### Simulation modeling

Transport phenomena of MENPs inside the cell via NEP was simulated in a commercial finite element method (FEM) solver, COMSOL Multiphysics 4.3b, by using three different modules. First, *Electric Currents* application mode of the *AC/DC* module was used to solve for the induced transmembrane voltage (ITV). Then, the differential equation characterizing the cell membrane pore formation dynamics was incorporated and solved in COMSOL by making use of the *Weak Form Boundary PDE* application mode under the *PDE* module. Finally, transport mechanisms of MENPs inside the cell under the presence of external field were successfully implemented in COMSOL using *Transport of Diluted Species* module. We used *Time Dependent Study* to couple all three modules and update the results at each time step of the simulation.

### Statistics

All the experiments reported in this research were conducted at least three times in duplicate and values were expressed as means ± standard deviation (SD). An unpaired student t-test was performed and a *p* values ≤ 0:05 were regarded as significant.

## Additional Information

**How to cite this article:** Kaushik, A. *et al*. Investigation of ac-magnetic field stimulated nanoelectroporation of magneto-electric nano-drug-carrier inside CNS cells. *Sci. Rep.* **7**, 45663; doi: 10.1038/srep45663 (2017).

**Publisher's note:** Springer Nature remains neutral with regard to jurisdictional claims in published maps and institutional affiliations.

## Figures and Tables

**Table 1:** Simulation model parameters.

Definition	Symbol	Value & Ref.
Cell radius	R	8 μm
Cell membrance thickness	h	5 nm^40^,^41^,^42^,^43^
Cytoplasmic relative permittivity		80^38^,^39^
Relative permittivity in the extracellular medium		80^38^,^39^
Cytoplasmic electric conductivity	σ_I_	0.25 S/m^38^
Extracellular electric conductivity	σ_E_	0.01 S/m^38^
Diffusion coefficient of MENPs	D_p_	250 μm^2^/s
Effective charge number for MENPs	Z_eff_	+2
Creation rate coefficient	*α*	1 × 10^9^ m^−2^s^−1^ 38,39,40,41,43
Pore creation rate	q	2.46^38^,^39^,^40^,^41^,^43^
Equilibrium pore density	N_0_	1.5 × 10^9^ m^−2^ 38,39
Characteristic voltage of electroporation	V_ep_	170 mV^39^
Single pore radius	R_p_	0.76 nm^38^,^39^,^43^
Conductivity of a single pore	σ_p_	(σ_E_ − σ_I_)/ln(σ_E_/σ_I_)^38^
Cell membrane passive electric conductivity	σ_m0_	2 × 10^−7^ S/m^38^,^39^
Cell membrane relative permittivity	ε_m_	5^39^,^43^
Elementary charge	e_0_	1.6 × 10^−19^C^38^,^39^,^40^,^41^,^43^
Boltzmann constant	k	1.38 × 10^−23^ JK^−1^ 38
Temperature	T	298 K^39^,^41^
External applied electric field strength	E	600 V/cm
Electric pulse duration		10 ms
Initial concentration of MENPs inside the cell		0 M
Initial concentration of MENPs outside the cell		1 mM
